# Medical students: They’re not just little doctors! Impact of an online group-coaching program on medical student well-being: A randomized clinical trial

**DOI:** 10.1371/journal.pone.0328546

**Published:** 2025-08-12

**Authors:** Adrienne Mann, Tyra Fainstad, Ivy Sullivan, Ethan M. Ritz, Jeffrey R. SooHoo, Hilit F. Mechaber, Ami Shah

**Affiliations:** 1 University of Colorado Division of Hospital Medicine, Department of Medicine, Anschutz Medical Campus, Aurora, Colorado, United States of America; 2 Veterans’ Health Administration, Eastern CO Health Care System, Aurora, Colorado, United States of America; 3 University of Colorado Division of General Internal Medicine, Department of Medicine, Anschutz Medical Campus, Aurora, Colorado United States of America; 4 Rush Medical School, Rush University Medical Center, Chicago, Illinois, United States of America; 5 Rush Biostatistics Core, Rush University Medical Center, Chicago, Illinois, United States of America; 6 University of Colorado, Department of Ophthalmology, Anschutz Medical Campus, Aurora, Colorado, United States of America; 7 University of Miami Miller School of Medicine, Department of Medical Education, Miami, Florida, United States of America; 8 Division of Pediatric Surgery, Department of Surgery, Rush University Medical Center, Chicago, Illinois, United States of America; University of Science and Technology of Fujairah, YEMEN

## Abstract

**Introduction:**

Physician burnout begins in medical school. Professional coaching can improve physician well-being, but generalizable evidence in medical students is lacking. We aim to evaluate a coaching program in a national sample of students.

**Materials and methods:**

A randomized clinical trial assessing a four-month, web-based, group coaching program was conducted between September 1, 2023, and December 31, 2023, among medical students from seven institutions. The primary outcome was burnout measured using the Maslach Burnout Inventory. Secondary outcomes included impostor syndrome, moral injury, self-compassion, and flourishing. A linear mixed effect model analysis was performed on an intent-to-treat basis.

**Results:**

Among the 390 participating students (mean [SD] age, 25.3 [2.38]), 197 were randomized to the intervention group. There were no significant post-coaching differences in burnout, moral injury or impostor syndrome between groups. After the coaching intervention, the intervention group had significantly greater self-compassion with an absolute difference of 3.00 points (SE = 1.29; 95% CI, 0.48 to 5.53 points; P = 0.021)), and significantly higher flourishing scores, with an absolute difference of 0.45 points (SE = 0.23; 95% CI, 0.01 to 0.90 points; *P* = 0.048) compared to the control.

**Conclusion:**

Web-based group coaching did not have an impact on measures of distress in medical students, though did improve well-being outcomes including self-compassion and flourishing.

**Trial Registration:** ClinicalTrials.gov Identifier: NCT05822375

## Introduction

Physician well-being in the post-pandemic era has faced a rapidly changing landscape, particularly for medical students. [1] The COVID-19 pandemic reshaped healthcare delivery and education, potentially altering how burnout affects physicians and trainees. [2] Notably, burnout rates among medical students have not worsened since the pandemic, and in many studies improved. [3–6] An early-pandemic study revealed that while burnout was decreasing, stress and isolation levels were higher. [6] A mid-pandemic study confirmed higher loneliness and career uncertainty. [5] Additionally, recent changes in assessment, such as pass/fail grading of the United States Medical Licensing Exam (USMLE) Step 1 and more prevalent pass/fail grading in clinical rotations have introduced various impacts, including an emphasis on research and extracurricular productivity, which may add to stress. [7] In this changing landscape, broader literature on effective well-being interventions in undergraduate medical education (UME) remains underexplored.

One promising intervention to address these challenges is professional coaching. Coaching is a tool to help learners by highlighting insights into assumptions, perceptions, and behaviors. [8,9] Coaching differs from therapy in that it does not diagnose or treat but instead uses inquiry and metacognition to foster motivation and monitor self-progress. [8,9] Evidence supporting coaching in medical students primarily focuses on one-on-one sessions led by non-certified faculty coaches, which can be expensive, time-consuming, and difficult to maintain reproducibility and validity. [10–13] To address these limitations, the previously developed Better Together Physician Coaching (“Better Together” or “BT”) program was refined for students, and implemented in seven medical schools. [14,15] Building on previous successes in improving well-being among physician trainees and faculty physicians, [14,15] this multi-site randomized controlled trial (RCT) aims to evaluate the effectiveness of the four month online group coaching program among a national sample of medical students.

### Theoretical framework

Better Together was originally developed for the graduate medical education population by two professional physician coaches (T.F. and A.M.). BT includes regularly scheduled group coaching calls delivered by trained volunteer physician certified coaches (See Supplement 1) who employ methodology based on cognitive behavioral coaching, [16] acceptance and commitment therapy, [17] and self-determination theory (SDT). [18] The coaching calls align with a digital 16-week curriculum specifically developed using SDT. SDT is a motivational theory that posits that a self-determined orientation is created from three ingredients: autonomy, competence, and relatedness and creates positive performance and well-being outcomes. [19,20] Each of the 16 weekly concepts is grounded in one of the core tenets of SDT: autonomy, competence, and relatedness (See Supplement 1 for more details on weekly themes and curricular content). Live and written coaching delivered in BT also employs SDT by emphasizing awareness, community and agency through a thought-based approach examining perceived experiences, belief systems, emotional regulation, and professional identity formation, ultimately fostering self-trust and emotional agility [9,21].

## Materials and methods

### Trial oversight

This RCT follows the Consolidated Standards of Reporting Trials (CONSORT) [22] reporting guideline and was approved by the University of Colorado institutional review board (COMIRB #23-0651, approved April 21^st^ 2023). The study was conducted from September 1, 2023, to December 31, 2023, at seven US medical schools of different geographic locations and sizes including schools that utilized academic, county, Veterans Health Administration, and community-based hospitals and clinics for student rotations (**S1 Table**). Recruitment emails were initially sent to medical school leadership to explore interest in participating and phone or video conference calls were held to confirm partnership and participation of each school in this study. Data were collected and managed with The University of Colorado Research Electronic Data Capture (REDCap). Participating sites could not access identifiable data.

### Participants and trial groups

**Fig 1:**
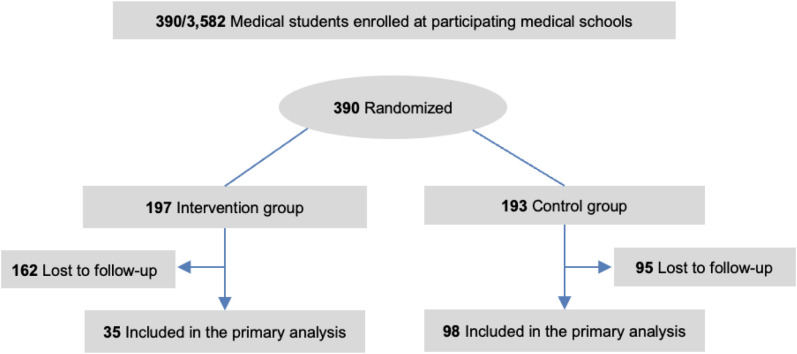
Study design.

At most participating institutions, all enrolled medical students were eligible to participate in the study regardless of training year. Two schools (University of Miami and University of Colorado) chose to offer BT to students in only one class (the M2 and M4 classes respectively) with the intent of following one cohort to assess impact specifically on that year because they were undergoing changes to their curriculum and wanted to avoid confounding information. All eligible students were recruited from July 1, 2023, through August 15^th^, 2023, with through a series of three emails to an electronic mailing list. Enrollment was voluntary, and all participants provided written informed consent. All students who volunteered into the study were enrolled. After enrollment, participants were randomly assigned to the intervention (access to online group coaching) or control group (no access to BT during the study). This was a two-arm, parallel-group randomized controlled trial. Participants were assigned in a 1:1 ratio to either the intervention or control group using a computer-generated algorithm embedded in the REDCap system. Allocation was concealed from investigators until assignment, and randomization was stratified by medical school year and site (**Fig 1**). Intervention participants were not given any extra time and carried the same preclinical/clinical schedules as control participants. All participants were offered baseline and 4-month (end of coaching intervention) surveys with self-reported demographics and validated tools measuring well-being. The control group was offered the coaching intervention after the study and was told they were on a “waitlist” for coaching during the study. To attempt to account for any benefits that may occur from student expectations rather than the coaching intervention, all participants were emailed alternative online wellbeing resources before the study intervention began.

### Intervention: Better together

Better Together Physician Coaching (“BT”) is a 4-month, digital group-coaching program developed by two professional physician coaches (TF and AM) shown to improve measures of wellbeing and distress both immediately and at 12 months after coaching ends in physicians and physician trainees. [14,15,21] BT is delivered by a cohort of physician coaches, all certified by The Life Coach School.^™^ Coach selection, onboarding and facets of the BT curriculum are described in the national physician trainee trial. [14] Logistically, the BT curriculum was developed using foundational theory of user engagement from Short et al (self-monitoring, reminders, and aesthetics), [14,15,23] and the Cole-Lewis framework for behavior change (modular, course-like, weekly content introduction). [14,15,24] Participants of the program are offered access to the following services which are located on a members-only, password-protected website: (1) live group coaching calls via video teleconference (Zoom Video Communications), (2) unlimited anonymous written coaching, and (3) weekly self-study modules on pertinent topics. The group sessions are recorded so participants can listen asynchronously, at a time that works for them via private podcast. [14,15] The program required about five hours per week of total coach time. BT was originally developed for a graduate medical education audience; however, this version was adapted slightly for medical students. Specifically, the core curriculum content remained the same because the investigators felt the topics were pertinent to students, however the cases and examples were adapted to include situations relevant to students. More information on the BT framework, modalities, and curriculum can be found in Supporting Information (**S1 Appendix**).

### Primary outcome

#### Burnout.

The 22-item Maslach Burnout Inventory (MBI) [25] is considered the benchmark standard to measure burnout and was used under license. The MBI contains 3 subscales: emotional exhaustion (EE; score range, 0–54), where a higher score indicates worse EE; depersonalization (DP; score range, 0–30), where a higher score indicates worse DP; and personal accomplishment (PA; score range, 0–48), where a higher score indicates better PA. We used established threshold definitions of high EE (subscale score ≥27), high DP (subscale score ≥10), and low PA (subscale score ≤33), and considered those with high EE or DP to have at least 1 manifestation of burnout and meet the definition for burnout. [26,27]

### Secondary outcomes

#### Self-compassion.

The Neff Self-Compassion Scale – Short Form (SCS-SF) is a 12-item instrument with a total score range of 12–60, where higher scores indicate greater self-compassion [28]. Although some studies categorize scores as low (12–30), moderate (30–42), and high (42–60), these cutoffs are not formally standardized and may vary depending on the population studied.

#### Flourishing.

Flourishing was evaluated with the Secure Flourishing Index (SFI), [29] which recognizes six domains: (a) happiness and satisfaction, (b) mental and physical health, (c) meaning and purpose, (d) character and virtue, (e) social relationships, and (f) financial and material stability. The scale contains 12 items, ranging from 0 to 10 with higher scores indicating greater flourishing. The SFI score is reported as an average (rather than sum) so the total ranges from 0 to 12.

#### Impostor syndrome.

The Young Impostor Syndrome Scale (YISS) is an 8-item instrument with yes-or-no scoring and a range of 0–8. [30] A score of 5 or greater indicates the presence of impostor syndrome.

#### Moral Injury.

The Moral Injury Symptom Scale–Healthcare Professionals (MISS-HP) is a 10-item instrument with scores ranging from 10 to 100. [31] Higher scores indicate greater moral injury.

### Statistical analysis

A sample size of 400 was chosen for the rigorous size powered to find a difference in our population, and considering a conservative estimate of the coaching program’s capacity to provide the intervention to 200 students at a time. Assuming a conservative assumption of zero correlation between 2 measurements for the same individual, with 80% power (α = .05, 2-sided), a mean (SD) standardized effect size of 0.8 (1.0) was detectable. With the use of SDs for the MBI components from prior studies of this intervention, we felt reassured in ability to detect a difference in mean (SD) values between the groups for all burnout components. [14,15]

Descriptive statistics were calculated for the overall cohort and stratified by intervention group. Comparisons between groups were conducted using t-tests or Wilcoxon rank-sum tests for continuous variables, and Fisher’s exact or chi-square (χ²) tests for categorical variables. Baseline characteristics were also compared between participants who completed the final survey and those who did not.

An intent-to-treat analysis was performed on all participants regardless of postsurvey completion. We used linear mixed-effects regression models to evaluate the effect of the intervention on each continuous outcome variable. The models included fixed effects for time (pre vs post), group (intervention vs control), and the interaction of time and group, with a random intercept for each participant to account for within-subject correlation over time estimated using restricted maximum likelihood. Mean change from baseline within each group and the difference in mean change between groups and their 95% CIs were reported. Linear mixed-effects regression models were used for EE, PA, DP, YISS, MISS-HP, SCS, SFI.

All *P* values were derived from two-sided hypothesis tests, with statistical significance evaluated at the α = .05 threshold. Analyses were conducted using R statistical software (version 4.2.3, R Project for Statistical Computing).

## Results

### Participants

A total of 390 medical students enrolled in the study and completed a baseline survey. Of those, 197 were randomized to the intervention and 193 were randomized to the control group (**Fig 1**). There were no significant baseline differences in demographics or outcome scores between groups (Table 1). The average age [SD] was 25.3 [2.58] years), 269 (70%) self-reported their identity as women, and 170 (45%) self-reported their race as White. There were 102 students (26%) in medical student year (M) 1, 105 (27%) in M2, 83 (21%) in M3, and 100 (26%) in M4 or beyond.

**Table 1:** Baseline Characteristics of All Participants.

		Patricipants No. (%)			
Variable	N completed	Overall, N = 390^*1*^	Waitlist, N = 193^*1*^	Intervention, N = 197^*1*^	p-value^*2*^
Surveys Completed	390				<0.001
1		257 (66%)	95 (49%)	162 (82%)	
2		133 (34%)	98 (51%)	35 (18%)	
Age	382	25.33 (2.58)	25.33 (2.61)	25.32 (2.56)	>0.9
Year in Medical School	390				0.9
1		102 (26%)	53 (27%)	49 (25%)	
2		105 (27%)	53 (27%)	52 (26%)	
3		83 (21%)	38 (20%)	45 (23%)	
4 or beyond		100 (26%)	49 (25%)	51 (26%)	
Gender Identity	382				0.5
Cisgender woman		269 (70%)	134 (71%)	135 (70%)	
Cisgender man		100 (26%)	51 (27%)	49 (25%)	
Trans male		3 (0.8%)	0 (0%)	3 (1.6%)	
Non-binary/third gender		9 (2.4%)	4 (2.1%)	5 (2.6%)	
Genderqueer		1 (0.3%)	0 (0%)	1 (0.5%)	
Race/Ethnicity	374				0.6
Asian		97 (26%)	42 (23%)	55 (29%)	
Black or African American		36 (9.6%)	15 (8.1%)	21 (11%)	
Hispanic or Latinx		16 (4.3%)	8 (4.3%)	8 (4.2%)	
Middle Eastern or North African		8 (2.1%)	4 (2.2%)	4 (2.1%)	
White		170 (45%)	90 (49%)	80 (42%)	
Multiple		47 (13%)	26 (14%)	21 (11%)	
Characteristic					
Burnout Emotional Exhaustion score mean (SD)	379	26 (11)	27 (10)	26 (11)	0.5
Emotional Exhaustion ≥ 27	379	192 (51%)	94 (49%)	98 (52%)	0.6
Burnout Depersonalization score mean (SD)	367	7.6 (5.6)	7.8 (5.7)	7.4 (5.5)	0.5
Depersonalization ≥ 10	367	123 (34%)	70 (38%)	53 (29%)	0.055
Emotional Exhaustion ≥ 27 or Depersonalization ≥ 10	375	227 (61%)	117 (62%)	110 (59%)	0.6
Burnout Personal Accomplishment score mean (SD)	374	34 (8)	33 (8)	34 (8)	0.2
Personal Accomplishment ≤ 33	374	172 (46%)	99 (53%)	73 (39%)	0.009
Self-Compassion Scale score mean (SD)	370	33 (8)	33 (8)	33 (8)	0.4
Young Imposter Scale score mean (SD)	369	4.94 (2.14)	4.88 (2.16)	5.00 (2.13)	0.6
Young Imposter Scale ≥ 5	369	215 (58%)	107 (58%)	108 (58%)	>0.9
Moral Injury Symptom Scale score mean (SD)	354	59 (21)	59 (21)	60 (21)	0.9
Secure Flourishing Index score mean (SD)	361	6.42 (1.31)	6.43 (1.27)	6.41 (1.35)	0.9

^*1*^ Mean (SD); n (%).

^*2*^ Two Sample t-test; Pearson’s Chi-squared test.

At baseline, the mean (SD) score was 26 (11) for EE, 7.6 (5.6) for DP, and 34 (8) for PA. Out of the 375 students who completed the full MBI, 227 (61%) met criteria for burnout. Of the 369 who completed the YIS, 215 (58%) scored positively for impostor syndrome. The mean (SD) baseline scores were high at 59 (21) for moral injury, low-moderate at 33 (8) for self-compassion, and moderate 6.42 (1.31) for flourishing.

Just over a third (133, 34%) of students completed the post-survey, with higher percentage of those being control compared to intervention participants (51% vs. 18%), however post-survey respondents and nonrespondents did not differ otherwise by race, gender identity, or year in medical school (**S2 Table**).

### Primary outcome

There were no significant improvements in burnout between groups. Specifically, intervention group mean EE change was −0.94 points (SE = 1.53, 95% CI, −3.93 to 2.05 *P* = 0.539), while the control group was −0.83 (SE = 0.92, CI, −2.63 to 0.96, *P =* 0.364*)* with an absolute between group difference of −0.10 (SE = 1.78. CI, −3.59 to 3.38, *P =* 0.953). The DP intervention group mean change was −0.68 (SE = 0.84, 95% CI, −2.33 to 0.98, *P* = 0.422), while the control group mean change was 0.02 (SE = 0.51,95% CI, 0.99 to 1.02, *P* = 0.973*)* with an absolute between group difference of −0.70 (SE = 0.99, CI, −2.63 to 1.24, *P = *0.482). The PA intervention group mean change was 2.05 (SE = 1.07, 95% CI, −0.05 to 4.16, *P* = 0.058), while the control group mean change was 0.03 (SE = 0.64, 95% CI, −1.22 to 1.28, *P* = 0.964*)* with an absolute between group difference of 2.02 (SE = 1.25, CI, −0.42 to 4.47, *P = *0.107).

### Secondary outcomes

There was a significant improvement in self-compassion (Table 2). The mean change on the Self-Compassion Scale–Short Form was 3.03 points (SE = 1.11; 95% CI, 0.86 to 5.20; *P* = 0.007) for the intervention group, while the control group did not show a statistically significant change with a point difference of 0.02 points (SE = 0.66; 95% CI, −1.27 to 1.31 points; *P* = 0.97). The between-group absolute difference was 3.00 points (SE = 1.29; 95% CI, 0.48 to 5.53 points; *P* = 0.021).

**Table 2:** Mean Change in Response from baseline visit, Established from Linear Mixed Effect Models.

Outcome	Waitlist Δ (95% CI)	SE	P	Intervention Δ (95% CI)	SE	P	Absolute difference in Δ: (95% CI)	SE	P
Burnout Emotional Exhaustion	−0.83 (−2.63, 0.96)	0.92	0.364	−0.94 (−3.93, 2.05)	1.53	0.539	−0.10 (−3.59, 3.38)	1.78	0.953
Burnout Depersonalization	0.02 (−0.99, 1.02)	0.51	0.973	−0.68 (−2.33, 0.98)	0.84	0.422	−0.70 (−2.63, 1.24)	0.99	0.482
Burnout Personal Accomplishment	0.03 (−1.22, 1.28)	0.64	0.964	2.05 (−0.05, 4.16)	1.07	0.058	2.02 (−0.42, 4.47)	1.25	0.107
Self-Compassion	0.02 (−1.27, 1.31)	0.66	0.972	3.03 (0.86, 5.20)	1.11	0.007	3.00 (0.48, 5.53)	1.29	0.021
Impostor Syndrome	0.23 (−0.10, 0.56)	0.17	0.176	−0.13 (−0.68, 0.43)	0.28	0.655	−0.36 (−1.00, 0.29)	0.33	0.28
Moral Injury	0.91 (−2.62, 4.44)	1.80	0.613	−4.71 (−10.5, 1.10)	2.96	0.113	−5.63 (−12.41, 1.17)	3.46	0.107
Flourishing	−0.05 (−0.29, 0.18)	0.12	0.651	0.40 (0.02, 0.78)	0.19	0.048	0.45 (0.01, 0.90)	0.23	0.048

**Fig 2:**
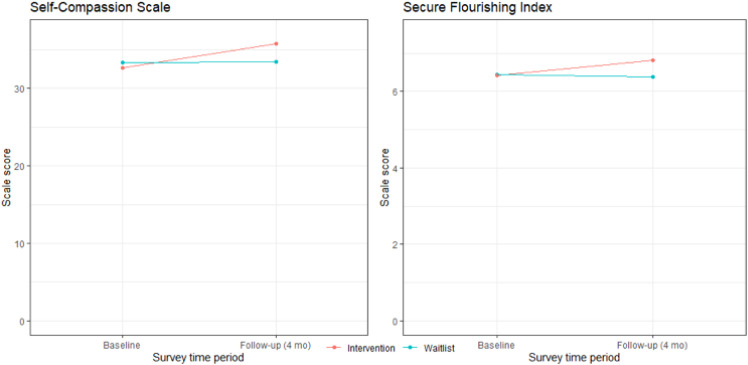
Mean Change in Well-being Outcomes from Baseline, Estimated From Linear Mixed-Effects Models.

There was also a significant improvement in flourishing (Table 2). The mean change on the Secure Flourishing Index improved for the intervention group by 0.40 points (SE = 0.19; 95% CI, 0.02 to 0.78; *P = 0.048*) but did not significantly change for the control group at −0.05 points (SE = 0.12; 95% CI, −0.29–0.18; *P = *0.651). The between-group absolute difference was 0.45 points (SE = 0.23; 95% CI, 0.01 to 0.90 points; *P* = 0.048) (**Fig 2**).

There were no significant improvements in impostor syndrome or moral injury between groups after coaching. The intervention group mean change in impostor syndrome was −0.13 points (SE = 0.28, 95% CI, −0.68 to 0.43; *P* = 0.28), while the control group was 0.23 (SE = 0.17, 95% CI, −0.10 to 0.56, *P = 0.176)* with an absolute between group difference of −0.36 (SE = 0.33, CI, −1.00 to 0.29, *P =* 0.28). The moral injury intervention group mean change was −4.71 (SE = 2.96, 95% CI, −10.51 to 1.10, *P* = 0.11), while the control group mean change was 0.91 (SE = 1.80, CI, −2.62 to 4.44, *P = 0.613)* with an absolute between group difference of −5.63 (SE = 3.46, CI 95%, −12.41 to 1.17, *P =* 0.107).

## Discussion

In this national RCT, students who received online group coaching over 4 months had improvements in well-being (self-compassion and flourishing), although not in measures of distress (burnout, impostor syndrome, or moral injury). We have previously demonstrated that BT coaching reduced burnout and all other measured distress markers among graduate medical education (GME) physician trainees. [14,15] In this study however, improvement in burnout and other markers of distress was *not* seen among medical students. This finding is likely multifactorial. First, the nature and content of the coaching program was originally designed for GME trainees and later for practicing clinicians. While we did adapt the examples and cases to make them relevant to student scenarios, we did not significantly change the overall weekly themes/objectives (i.e., “impostor syndrome” or “challenging feedback”) out of a belief that students faced similar obstacles. The main stressors that medical students experience (loneliness, exam frequency, financial burdens, career uncertainty), [5,32,33] however, do differ from physician stressors (challenges of documentation and practice management, changing roles in the workplace). [14,27,34] The BT weekly content therefore may have been less pertinent for students, explaining the lower impact in this population. This has prompted a qualitative study to better understand the needs and stressors of medical students in the coaching space which will inform future iterations of BT. Additionally, burnout may not be best measures of overall medical student distress, in light of the recent data showing burnout has not worsened. [3–6] A study by Chakladar et al, demonstrated that after the onset of the COVID-19 pandemic, medical students experience distress predominantly in the form of isolation, anxiety, and uncertainty about the future while overall burnout had decreased. [35] This points to a need for interventions that target additional facets of distress and prompts additional research into understanding these needs. In addition to potential curricular misfit, other explanations may account for the lack of improvement in distress measures. It is possible that the duration of the 4-month intervention was insufficient to impact entrenched forms of distress such as burnout or moral injury, which often develop over extended periods, although previous studies of BT suggest that the duration is sufficient in physicians and physician trainees required to shift these outcomes. [14,15] Moreover, medical students in our study had relatively low baseline levels of burnout and impostor syndrome compared to national norms for their physician counterparts, raising the possibility of either floor effects limiting our ability to detect change, or of measuring the wrong domains of distress. [35] Future studies could consider alternative timing, duration, or tools to better capture early signs of distress mitigation.

We did find significant improvements in the well-being metrics of self-compassion and flourishing. Previous work in the general adult population has shown that even a 0.1 point improvement in the SCS score is clinically meaningful, making our 3 point increase clinically significant. [36] A core focus of BT coaching is grounded in self-compassion, perhaps contributing to the largest improvements lying in this measure. [15,21] Low self-compassion is associated with eventual worsening burnout and a habit of deferring self-care. [37] A self-compassion deficit seen in physicians and trainees is likely multifactorial, but stems from a culture which often asks them to do more with less and normalizes self-sacrifice. [38] Furthermore, medical training often responds to mistakes with shame and blame which frame self-compassion as unnecessary and even counterproductive. [37,39] Reframing this belief and providing tools to create and protect self-compassion is foundational to BT. Given that self-compassion is a learnable skill, [40,41] it will be important to examine how change in self-compassion leads to well-being to fully understand this outcome. Among medical students and health care providers of other career stages and disciplines, self-compassion is significantly associated with lower burnout as well as increased well-being and compassion for others. [42–44] Research in college students and adults shows self-compassion has positive associations with happiness, optimism, intrinsic motivation, and negative associations with outcomes such as depression, anxiety, maladaptive perfectionism, self-criticism, and fear of failure — outcomes of high relevance to medical students. [45–47]

We also found a statistically significant, though modest improvement in flourishing. There is a lack of an agreed-upon benchmark to define a clinically meaningful change in flourishing, though prior studies have shown that even a sub-1 point negative change in flourishing in physician trainees was associated with lower quality of life, high emotional exhaustion, lower empathic concern. [48] Flourishing in physician trainees is lower on average than the general population, [29,49] Our study confirms this finding is also consistent in medical students. Recently, a model of precision well-being in medical education has been described, where medical students were classified into phenotypes based on their psychological distress and flourishing. [50] While 46% were “Healthy Flourishers” described as thriving, a low but concerningly real 1.3% of this “healthy” group endorsed suicidal ideation. One third identified as “Getting By” and endorsed higher depressive and anxiety symptoms with, 11% of this group reporting suicidal ideation or thoughts of self-harm. While these students are managing, they could benefit from well-being interventions such as coaching to holistically promote flourishing.

The improvements in self-compassion and flourishing, though modest in absolute terms, have meaningful implications. A 3-point increase in the Self-Compassion Scale (SCS) exceeds the 0.1–0.2 point threshold considered clinically significant in prior studies. [36] While our flourishing effect size was smaller, prior work has shown that even sub-1 point decreases in flourishing among trainees are associated with increased emotional exhaustion and lower quality of life. [48] Thus, even small improvements may reflect important protective effects, especially in a preventive, upstream intervention like this. These findings are consistent with prior literature showing self-compassion training improves resilience, emotional regulation, and interpersonal functioning—skills particularly relevant to medical students navigating high-pressure environments. [41,44,45] Future research should explore how early gains in self-compassion and flourishing may buffer against downstream distress, and whether integrating coaching earlier or with higher frequency may enhance these effects.

Coaching offers a way to deliver precision well-being to a large group of medical students. Group coaching studies are growing and offer scalability, low-cost and feasibility compared with individual coaching programs (especially if digital) and can therefore democratize coaching. [9] Another benefit is the development of community and the benefit of having challenges normalized among peers. In a recent advisory, the US Surgeon General strongly recommends integrating social connection into wellness programs. [51] The scalability, accessibility, and community-building aspects of BT offer unique advantages, particularly for students in underserved or geographically isolated areas. This approach, grounded in the learner’s experience, provides an adaptable framework that can be tailored to address the diverse needs of medical students. The results support the growing body of evidence that coaching, especially in group formats, can provide access to mental health resources in medical education.

Here, we show that a group coaching model that is successful in practicing physicians may also help medical students thrive, though we have work to do to understand how to also improve measures of distress. The evidence and theoretical frameworks that informed Better Together include facets around autonomy, treatment of self, common humanity, overidentification and character/virtue for a more comprehensive picture of thriving. [17,20,21] BT was created fundamentally using SDT given its well described impact in medical education as well as on well-being, including burnout. [19,20,52] As described above, we included SDT foci of autonomy, mastery, and belonging throughout the curriculum and live coaching. One significant difference for medical students compared to practicing physicians is they often lack one or more of these three necessary components of self-determination. [52] Students are subject to a relatively fixed schedule and are often not able to provide input or control their day, significantly limiting their autonomy. Additionally, their autonomy at work is limited compared to their physician counterparts. As learners, they have not mastered certain aspects of medical knowledge or procedural skills and may also question how they belong or fit into the culture of medicine. It may be that these different stressors affecting medical students require different interventions or a modified version of BT to address measures of distress among medical students.

## Limitations

Voluntary participation may have created selection bias at the individual and institutional level. Institutional buy-in was required which potentially favors sites more supportive of well-being initiatives. Similarly, participants may have either been more severely in need of well-being help, or more self-aware or receptive to coaching. We did not control for unmeasured confounders such as prior coaching experience, access to other wellness resources, or pre-existing mental health conditions that could influence outcomes. We did not use cluster randomization given our interest in individual over group-level changes in outcomes, and we wanted to avoid loss of statistical power. This study is unique in that the intervention was administered digitally by a centralized entity (not separately at each site as in more traditional clinical trials). Contamination between control and intervention participants at the same sites was possible, though we mitigated this through password protection of materials.

We had substantial loss to follow-up, which may have introduced bias limiting the generalizability of the findings. Control participants were significantly more likely to respond to the survey than intervention participants, perhaps due to email fatigue (the intervention group received 2 emails weekly), or control participants may have been more motivated in anticipation of receiving the intervention. Reassuringly, there were no significant baseline differences in demographics or outcome scores between groups, or between respondents and non-respondents. Still, those lost to follow-up may represent a group too overwhelmed to participate or, alternatively, those doing well and less inclined to re-engage.

Because we allowed participants to choose their level of anonymity and to use unique and different logins for each coaching call, downloaded podcast, and curriculum module, we were unable to measure engagement or dose-response to correlate it with outcomes. Additionally, the study team and participants could not be masked, and outcomes could have accrued in part from participant expectations despite providing both groups access to well-being resources as a plausible alternative.

Also, as stated above, the curriculum was initially designed for GME. Further research is needed to refine coaching programs for undergraduate medical students, particularly to better address their unique stressors. Despite its limitations, this study provides valuable insights into the potential for coaching to enhance the well-being of medical students and offers a pathway for future interventions.

## Conclusion

This large, multi-site randomized controlled trial demonstrates the potential for online group coaching to positively impact medical student well-being by improving self-compassion and flourishing, however it did not show significant effects on burnout, moral injury or impostor syndrome, illuminating an unmet need in medical students compared to physicians.

## Supporting information

Supplemental Information 1 Supplement 1 Table: Site Participation. Supplement 2 Table: Post-Test Non-responders vs. Responders. Supplement 1 Appendix: BT Coach Onboarding and Facets of BT program.
(DOCX)

Supplemental Information 2 Trial Protocol. (DOCX)

Supplemental Information 3 CONSORT Checklist. (DOC)
