# Supplementary material for: Medical students: They’re not just little doctors! Impact of an online group-coaching program on medical student well-being: A randomized clinical trial
Source: PLoS One. 2025 Aug 12;20(8):e0328546. doi: 10.1371/journal.pone.0328546 (PMC12342239; doi:10.1371/journal.pone.0328546)
Supplement: Supplemental Information 1 — Supplement 1 Table: Site Participation. Supplement 2 Table: Post-Test Non-responders vs. Responders. Supplement 1 Appendix: BT Coach Onboarding and Facets of BT program. (DOCX) [file pone.0328546.s001.docx]

**Supporting Information**

**S1 Table: Site Participation**

| Site (State) | # of Participants (% total) |
| --- | --- |
| Site a (University of Colorado School of Medicine) | 28 (7.2%) |
| Site B (Duke University School of Medicine) | 65 (16.6%) |
| Site C (Johns Hopkins University School of Medicine) | 66 (16.9%) |
| Site D (University of Kentucky College of Medicine) | 98 (25.1%) |
| Site E (Miller School of Medicine, University of Miami) | 17 (4.4%) |
| Site F (Warren Alpert Medical School of Brown University) | 79 (20.2%) |
| Site G (Yale University School of Medicine) | 37 (9.5%) |

**S2 Table:** Post-Test Non-responders vs. Responders


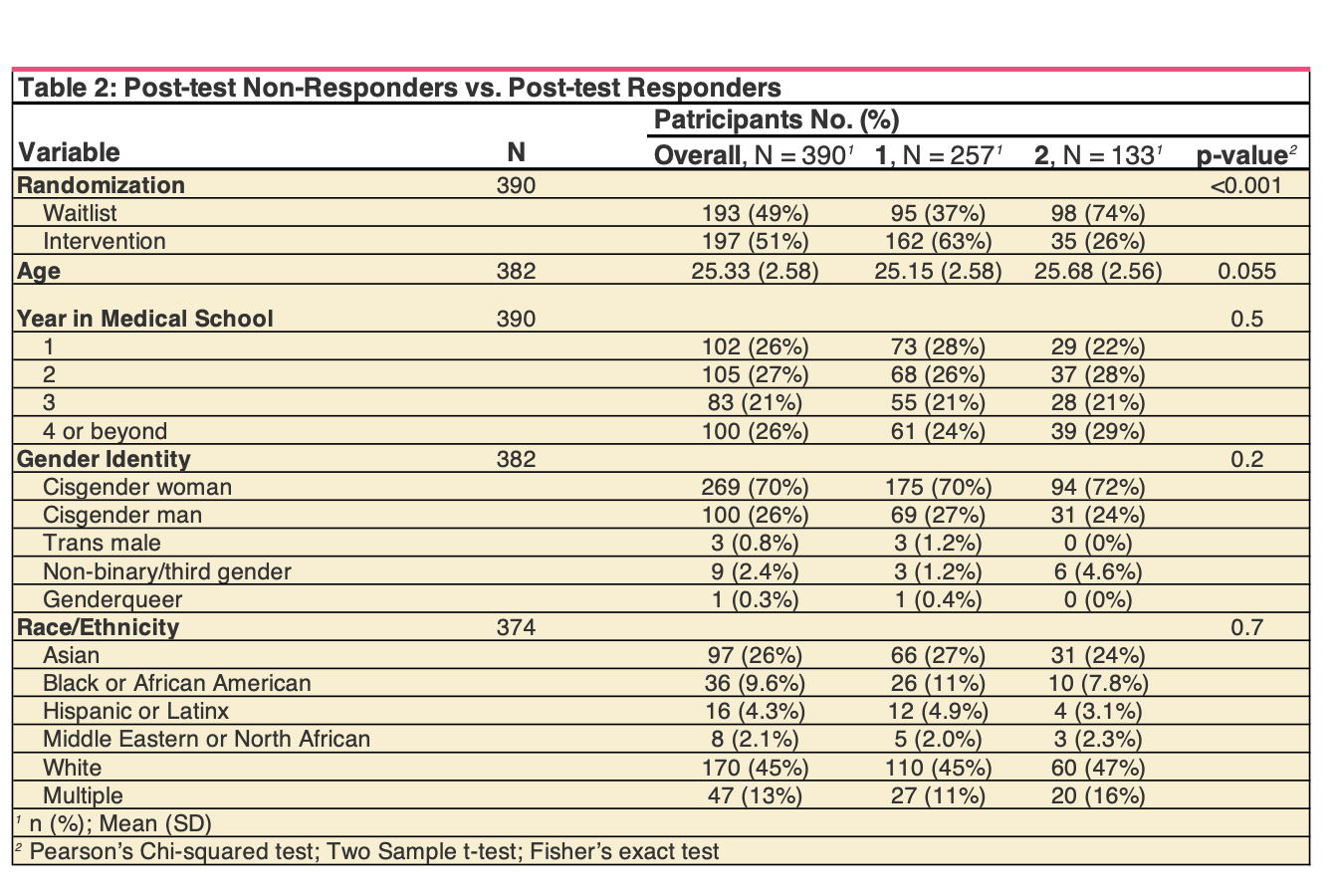


**S1 Appendix:**

**BT Coach Onboarding and Facets of BT program**

***Better Together Coach Onboarding Process:***

To deliver the program at scale we onboarded >20 physician coaches from across the country, all physicians and all certified through the Life Coach School (see #2 below). Though BT coaches have all been certified through LCS, none are affiliated with the LCS in an ongoing way, specifically, none are currently employees or teachers for LCS. The LCS was chosen for its thought based coaching methodology (rather than behavior based), technique is theory-informed, evidence-based, and algorithmic. Because of this, the coach who uses the LCS model delivers a reproducible and valid technique, which is not the case in many other programs. Additionally, most other coaching programs center on hour-long 1:1 coaching sessions, while LCS specifically trains to provide coaching in both written, and a virtual (zoom) group format as well as how to facilitate shorter sessions (10-15minuts), which are imperative to our group calls. Coach certification cost between $15,000-$20,000 for our coaches (prices have increased over time). Many of the BT coaches paid for this out of their own pocket (including AM and TF), however, some have been supported in full or part by their institutions. Each coach volunteered to host at least two coaching calls during the 4-month program on the Better Together platform with regular oversight and feedback from Drs. Mann and Fainstad to ensure internal reliability and quality of coaching. Though some of these coaches were faculty at participating sites, that was not a requirement. In the instance that a BT coach coincidentally supervised a participant in a clinical or educational setting, they recused themselves from any formal assessment to avoid potential conflict of interest.

To ensure quality and internal consistency of our coaches and coaching, we had the following criteria for each coach:

1) Must be a physician (MD or DO).

2) Must have graduated from the Life Coach School (LCS) certification program.

3) Must submit a sample coaching call which is reviewed by Drs. Mann and Fainstad, with provision of feedback with a standardized peer- observation feedback tool (available upon request). If their coaching sample was acceptable, the coach was invited to be added to our roster.

4) Must agree to receive self and peer-feedback on coaching. Once on our roster, coaches sign up to host coaching calls. These calls are observed intermittently with provision of feedback via a standardized peer-observation tool.

For ongoing fidelity/quality assurance of coaching, Drs. Fainstad and Mann observed and provided feedback for each coach at least once during the intervention and did this on a quarterly basis for each coach that works in BT in previous (local) iterations of this program. If a coach was not providing coaching consistent with the model and feedback framework, then underwent remediation with Drs. Fainstad and Mann until these metrics are met. All coaching calls were recorded and housed on a secure, members-only website.

***BT foundational framework:***

| Certified physician coaches | Many coaching interventions use non-physician coach consultants or rely on volunteer physician faculty with variable degrees of faculty development training rather than formal certification.[^11,19^](https://paperpile.com/c/sURvNY/2OKxI+TmUW) Utilization of physician coaches allowed for contextual understanding of challenges of medical training and offered relatability and credibility to participants. |
| --- | --- |
| Group Coaching | Delivering coaching on a group call offers the same impact for the individual being coached with the additional benefit for all observing who can apply lessons learned and gain a sense of normalization of vulnerability and community. |
| Flexibility | The asynchronous, multimodal content delivered by BT allows trainees to access material on-demand. The repository of recorded calls allows participants who miss a call to still reap the benefits. Having volunteer coaches across the country allowed us to offer a variety of call times to accommodate trainee schedule needs across 5 time zones. |
| Personalized Anonymity | Participants could choose how, when and whether to show up on any facet of BT. Group calls were held in a webinar format, so the audience was completely anonymous. If they chose to join as a panelist and be coached live, they decided on their visibility (camera on or off, use of their name, etc.). They also had the option to be coached live in written format using the chat function. Offering an asynchronous anonymous ask-for-coaching written forum offered those who felt too exposed on live calls a space to write in and receive coaching in that form as well. |
| Data-driven | The BT program is evidence-based, and we employ a “Plan-Do-Study-Act” (PDSA) cycle (an iterative, four-stage problem-solving method used for improving a process) after each coaching cohort in which we review the various coaching requests that arose in our participants and focus our curriculum and coach training around these areas specifically. The program is iteratively improved between each cycle based on participant feedback (eg: inclusion of a week on microaggressions and bias, and shortened length) |

***Modalities of the Better Together Coaching Program:***

*Live Coaching Calls.* The live coaching calls were facilitated by one of the certified physician coaches. Calls were hosted on the Zoom video-conferencing platform and were 60 minutes each. Getting coached on a live call was optional and voluntary: participants could raise their hand to request to be coached while the other participants on the call observed. Coaching on a call could be received anonymously through the chat, Q&A function or verbally. Call audio recordings were published on a private podcast accessible only to participants so they could listen to any calls they were not able to attend live. Coaching calls were scheduled at a variety of days and times to serve participants in different time zones and with variable schedules.

*“Ask-for-Coaching” Written Coaching.* The secure website housed an online forum where participants could anonymously request coaching via written format. Responses to written coaching requests were posted to the forum within 1-business day by TF or AM. This asynchronous format allowed access to coaching around-the-clock and provided a kinesthetic learning format for participants to engage with the coaching material. Ask-for-Coaching could be accessed at any point throughout the 4-month intervention, and the coachee was not identifiable to other participants.

*Worksheets and Webinars.* At the start of each month of content, worksheets were released which accompanied each weekly theme (i.e., receiving feedback, setting goals, adopting a growth mindset, or defining work-life balance). The worksheets facilitate metacognitive techniques of awareness and processing feelings. The worksheets were voluntary and meant for independent self-study. Short 5–10-minute webinar videos corresponding to weekly content were released at the start of each month. The webinars were chalk-talk style didactics given by AM or TF to introduce weekly themes. As they may be watched repeatedly, and at any time, the webinars served as an asynchronous component of the program that participants engaged with at their own pace.

***BT worksheet/webinar curriculum:***

Month 1: Coaching 101

Week 1: Metacognition: Introduction to the coaching model (metacognition, “autonomy” facet of SDT)

Week 2: Emotions: How to name and process an emotion (metacognition, “autonomy” facet of SDT)

Week 3: Defining your Purpose: using values to create meaning (metacognition, “autonomy” and “relatedness” facets of SDT)

Week 4: Living your Purpose at Work: finding your power to mitigate moral injury (metacognition, “autonomy” and “relatedness” facets of SDT)

Month 2: Feedback

Week 1: Growth Mindset (“autonomy” and “competency” facets of SDT)

Week 2: Welcoming Feedback: especially when it’s hard to hear (“autonomy” and “competency” facets of SDT)

Week 3: Bias and Microaggressions: accessing empowerment instead of burnout (“autonomy” and “relatedness” facets of SDT)

Week 4: Being your Best at Work: showing up for yourself even when it’s challenging (“autonomy” and “competency” facets of SDT)

Month 3: Imposter Syndrome

Week 1: Imposter Syndrome: getting to know your inner critic (“autonomy” and “relatedness” facets of SDT)

Week 2: Perfectionism: the stealthy self-saboteur (“autonomy” and “relatedness” facets of SDT)

Week 3: Approval Addiction: why people pleasing doesn’t work for you (“autonomy” and “relatedness” facets of SDT)

Week 4: How to change: reframe your inner critic (“autonomy” “competency” and “relatedness” facets of SDT)

Month 4: Launching into the New You

Week 1: Self-confidence: how it’s different from arrogance and task-confidence (“autonomy” and “relatedness” facets of SDT)

Week 2: Transitions: navigating through constant change (“autonomy” “competency” and “relatedness” facets of SDT)

Week 3: Self-Compassion: tapping into your inner wisdom (“autonomy” “competency” and “relatedness” facets of SDT)

Week 4: The New You- become your own best friend, for life (“autonomy” “competency” and “relatedness” facets of SDT)
