# Supplementary material for: Medical students: They’re not just little doctors! Impact of an online group-coaching program on medical student well-being: A randomized clinical trial
Source: PLoS One. 2025 Aug 12;20(8):e0328546. doi: 10.1371/journal.pone.0328546 (PMC12342239; doi:10.1371/journal.pone.0328546)
Supplement: Supplemental Information 2 — (DOCX) [file pone.0328546.s002.docx]

**Trial Protocol**

**Study Plan:**

**Brief Summary**

Better Together Physician Coaching ("Better Together", or "BT"), a 4-month, web-based positive psychology multimodal coaching program was built to decrease burnout in medical trainees. Here, the investigators seek to understand it's efficacy in University of Colorado School of Medicine (CU SOM) clinicians

Aim 1: Implement Better Together in undergraduate medical education settings for medical students Aim 2: Assess outcomes: primary: reduce burnout as measured by the Maslach Burnout Index (goal: 10% relative improvement), and secondary: self-compassion, imposter syndrome, flourishing, loneliness, and moral injury.

Aim 3: Advance the field of coaching for clinicians through innovation and dissemination of evidence-based approaches to medical student wellbeing.

**Detailed Description**

Burnout refers to feelings of exhaustion, negativism, and reduced personal efficacy resulting from chronic workplace stress. In healthcare, burnout leads to increased medical errors, poorer patient care and negatively affects professional development and retention. Burnout is a growing problem that begins early in medical training. Professional coaching is a metacognition tool with a sustainable positive effect on physician well-being but typically relies on expensive consultants or time-consuming faculty development, often making it infeasible for medical training programs to offer. To overcome this barrier, the investigators created Better Together Physician Coaching (BT) a 4-month coaching program for at the University of Colorado (CU). BT includes regular online group-coaching, written coaching, and weekly self-study modules delivered by physician life coaches (Co-PIs). In 2021, the investigators studied BT in a group of female-identifying resident trainees at CU and found that the program significantly improved burnout, imposter syndrome, and self-compassion. This finding supports previous data that life coaching is effective for physicians and physicians in training. The investigators initially focused on women since burnout affects women to a greater degree than their male counterparts, and may have long-lasting consequences on their careers, contributing to a "leaky pipeline" effect. The pilot randomized controlled trial (RCT) of 101 BT women participants demonstrated a statistically significant improvement in burnout, self-compassion, and imposter syndrome in the intervention group.

The investigators now seek to understand if the coaching program is also effective in medical students of all gender identities.

The hypothesis is that Better Together Physician Coaching ("Better Together", or "BT"), a 4-month, web-based positive psychology multimodal coaching program will result in decreased burnout in medical students.

Aim 1: Implement Better Together in the undergraduate medical student population

Aim 2: Assess outcomes: primary: reduce burnout as measured by the Maslach Burnout Index (goal: 10% relative improvement), and secondary: self-compassion, imposter syndrome, flourishing, loneliness, and moral injury.

Aim 3: Advance the field of coaching for clinicians through innovation and dissemination of evidence-based approaches to medical student wellbeing.

**Eligibility Criteria**

**Inclusion Criteria:** current medical students at one of the 5 US undergraduate medical education sites

**Exclusion Criteria:** non-medical students

**Ages Eligible for Study:** 18 Years to 65 Years (Adult, Older Adult )

**Sexes Eligible for Study:** All

**Primary Purpose:** Prevention
**Allocation:** Randomized
**Interventional Model:** Sequential Assignment

**Interventional Model Description:**
This study is a randomized controlled trial (RCT). All enrolled participants will complete a pretest baseline survey. After baseline data collection is completed, participants will be randomized into either a control or intervention group. The intervention group will receive the BT coaching program for four months (February 1, 2023 – May 31, 2023), while the control group will receive the coaching intervention after the study ends (a four-month delay (September 1, 2023 – December 31, 2023)).

At two time points, all participants will complete surveys assessing the following validated indices: burnout, imposter syndrome, self-compassion, moral injury, loneliness, and flourishing. Surveys will be administered at baseline (September 2023, T0) and post-intervention (January 2024, T1).

**Masking:** Single (Outcomes Assessor)
**Masking Description:** Data will be de-identified prior to analysis.

**Arms and Interventions**

| **Participant Group/Arm** | **Intervention/Treatment** |
| --- | --- |
| **Experimental: Intervention** | Will receive the BT coaching intervention for four months (September 1, 2023 – December 31, 2023). |
| **Behavioral: Better Together Physician Coaching** | Thought-based coaching integrating cognitive behavioral therapy (CBT), mindfulness-based awareness, acceptance and commitment therapy (ACT), nonattachment, and Socratic and Greek philosophy. BT delivers coaching via a 4-month web-based, group-coaching model, offering flexibility through multiple modalities: twice-weekly group coaching calls, unlimited anonymous written coaching, and weekly self-study modules on a secure website. |
| **No Intervention: Waitlist Control** | Will receive the coaching intervention following a four-month waitlist control period (February 1, 2024 – May 31, 2024). |

**Outcome Measures**

**Primary Outcome Measures**

| Outcome Measure | Measure Description | Time Frame |
| --- | --- | --- |
| **Burnout (Maslach Burnout Inventory, MBI)** | A 22-item measure assessing emotional exhaustion (EE), depersonalization (DP), and personal fulfillment (PF). Scores range from 0-6 per item. High burnout: EE ≥ 27, DP ≥ 10, PF < 33. Low burnout: EE ≤ 18, DP ≤ 5, PF ≥ 40. | Pretest before intervention and posttest after the 4-month intervention. |

**Secondary Outcome Measures**

| Outcome Measure | Measure Description | Time Frame |
| --- | --- | --- |
| **Self-Compassion (Neff's Self-Compassion Scale Short Form, SCS-SF)** | A 12-item scale measuring self-compassion, scored from 0-6 per item. Low: 1.0-2.49, Moderate: 2.5-3.5, High: 3.51-5.0. | Pretest before intervention and posttest after the 4-month intervention. |
| **Moral Injury (Moral Injury Symptom Scale for Health Professions, MISS-HP)** | A 10-item scale assessing moral injury, scored from 0-5 per item. Scores >35 (range: 10-100) indicate high moral injury symptoms. | Pretest before intervention and posttest after the 4-month intervention. |
| **Imposter Syndrome (Young's Imposter Syndrome Symptoms Scale, YISS)** | An 8-item yes/no measure of imposter syndrome. A score >5/8 is considered positive for imposter syndrome. | Pretest before intervention and posttest after the 4-month intervention. |
| **Flourishing (Secure Flourish Index, SFI)** | A 12-item scale assessing domains of well-being (happiness, health, meaning, character, relationships) plus stability and material security. Scores range from 0-120, reported as averages (0-10). | Pretest before intervention and posttest after the 4-month intervention. |
| **Loneliness (UCLA 3-item Loneliness Scale)** | A 3-item scale measuring loneliness, scored from 1-3 per item (1=Hardly ever, 2=Some of the time, 3=Often). Total scores range from 3-9, with higher scores indicating greater loneliness. | Pretest before intervention and posttest after the 4-month intervention. |
